# Aging and feature binding in visual working memory

**DOI:** 10.3389/fpsyg.2022.977565

**Published:** 2022-10-06

**Authors:** Alexandria Nicole Holcomb, Chiara Francesca Tagliabue, Veronica Mazza

**Affiliations:** Center for Mind/Brain Sciences (CIMeC), University of Trento, Trento, Italy

**Keywords:** feature-binding, visual working memory, cognitive aging, object representations, set-size

## Abstract

Older adults have reduced performance in visual working memory tasks in comparison to young adults, but the precipitators of the age-related impairment are not fully understood. The most common interpretation of this difference is that older adults are incapable of maintaining the same amount of object representations as young adults over short intervals (in line with the fixed-slot model of working memory). However, it has remained largely unexplored whether the age-related decline is only due to the number of representations that older individuals can retain in visual working memory, or whether the content of the representation(s) may have an effect as well (in line with the flexible-resource model of working memory). Feature binding studies represent an interesting research line to examine the content of older adults' representations. In this mini-review, we present the main results across feature binding studies in aging, as well as highlight the importance of manipulating both the representation content and number to have a stress test of the various models of working memory and their contribution to aging. Overall, feature binding studies, together with the simultaneous manipulation of set size, will allow us to better understand the nature of the age-related decline of visual working memory.

## Introduction

A typical cognitive impairment that follows the discourse of aging is reduced performance in working memory (WM) tasks (Salthouse et al., 1991; Park et al., 2002). This is generally observed as a lower accuracy in older adults (OAs) compared to young adults (YAs) with increasing sets of objects that need to be remembered (Jost et al., 2011; Sander et al., 2011; Tagliabue et al., 2019, 2022). Because of the set size manipulation, most studies have concluded that there is a reduction in the amount of object representations that OAs can hold in WM in comparison to YAs (Jost et al., 2011; Schwarzkopp et al., 2016; Tagliabue et al., 2020 but see Oberauer and Kliegl, 2010); this interpretation falls in line with the viewpoint of WM capacity as a fixed-slot model (Luck and Vogel, 1997; Cowan, 2001; see Adam et al., 2017 for more recent results), in which an individual's WM is set at a fixed number of representations, regardless of the content of the representations (Awh et al., 2007).

The fixed-slot model has been extensively researched in YAs, however met with inconsistent conclusions (see Alvarez and Cavanagh, 2004; Awh et al., 2007). An alternative viewpoint described WM in terms of functioning “flexibly,” in which limits vary as a function of the content of the object representation to maintain, and also at the detriment of less precise representations with increased amount of items to retain (Wilken and Ma, 2004; Bays and Husain, 2008). The issue of whether the nature of the representations (rather than their number) could be a key factor in understanding WM, is still under investigation in healthy young individuals (see Adam et al., 2017; Adam and Serences, 2019; Bouchacourt and Buschman, 2019), and has received little attention in the field of aging research. Specifically, the question remains as to what leads OAs to impaired performance in WM tasks: is it only the lower amount of WM representations retained, or are the representations that OAs hold in WM less precise than those of YAs? Or should one consider the interaction between the numerosity and the content of the representations?

These questions about the role of the content of the representation in the age-related WM decline, and its interaction with the number of representations, are the focus of the present mini-review. Accordingly, in the next sections we describe some of the extant studies on feature binding as a proxy to explore the age-related effect of the content of WM representation (in line with the view of WM as a flexible resource process; Wilken and Ma, 2004; Bays and Husain, 2008). It is well-known that the individual features of an object, such as color and shape are first processed separately, and then bound in order to form a complete representation in WM (Treisman, 1986; Schneegans and Bays, 2019). Additionally, the selection and retention of multiple features are more demanding than those of individual features (see Treisman and Gelade, 1980; Treisman, 1988, 1996; Schneegans and Bays, 2019). It is therefore possible that OA's WM impairments may arise as a result of impairments in the binding of multiple features in order to comprise a full representation in WM. Studies on age-related changes in feature binding have used this as a measure to probe the content of WM representations in aging, namely whether OAs demonstrate deficits in the ability to maintain bound object features over short intervals (see Allen et al., 2013 for a review). Finally, and in line with changing views of WM (Ma et al., 2014), we discuss the importance for future investigations to include both set size and feature binding manipulations (as it has been shown in studies on children, see Forsberg et al., 2022) to directly compare the flexible resource vs. fixed slot capacity accounts.

## Feature binding and working memory in aging

In this section, we describe the most relevant studies on WM examining the role of feature binding in aging (see Supplementary Table 1). An initial review on this topic (and effects in Alzheimer's Disease; AD) was provided by Allen et al. (2013). For the sake of clarity, we describe the two main types of binding that are typically examined in aging studies (i.e., object-to-location binding and within-object feature binding). We then illustrate those studies that found and those that did not find age-related effects on binding.

In the literature on aging and feature binding, two forms have been considered. The first form of binding is the ability to bind an object to its location. Although there are methodological variations throughout the majority of feature binding studies in aging, the standard paradigm of object-to-location binding presents participants with a small set of target objects. After a short delay, a test object is presented and the participant is required to report whether the object was previously presented (object-only identification), or alternatively, where in the spatial display the object had been presented (location-only identification), or both identity and location features (object-and-location identification) (Mitchell et al., 2000a). This latter condition is theorized to probe object-to-location binding as it measures not only individual feature recall (i.e., object-only and location-only), but also the bound representation of what the object was and where it was in the memorized display. Other investigations of binding have instead explored participants' ability to recall the bound representation of two surface features defining one object (typically color and shape), in comparison to trials in which the recall of single, individual features is required (within-object feature binding, e.g., Brown and Brockmole, 2010). In these studies, participants are presented with a display of shapes in different colors and after a delay, are probed with either individual feature recall of color trials, individual feature recall of shape trials, or both color and shape (constituting the bound representation of the two features; Brown and Brockmole, 2010).

Using a paradigm assessing object-to-location binding, Mitchell et al. (2000a) examined whether OAs showed disproportionate impairments in conditions in which subjects were probed to indicate either only the location of one of three previously presented object drawings (location trials), the object itself (object trials), or whether the exact test probe was presented in the same location as it had been in the previous memory array presentation (i.e., binding trials). The researchers found no age-related differences in location-only or object-only detection trials, but found an age-related decrement when both object and its location had to be recalled. In line with this study, Cowan et al. (2006) investigated OAs performance in a WM task probing memory for colored squares presented in different locations. In this task, in one condition one of the colored squares presented in a memory array changed in the test array to a different color (individual feature recall), while in the other condition, one of the colored squares presented in the memory array then changed to match another color present in the test array (binding condition). This binding condition should test the participants' ability to create bound representations of the object and its correct location. The researchers found an age-related effect in binding memory conditions when individual feature recall trials and binding trials were intermixed within the same block. The results of Mitchell et al. (2000a) and Cowan et al. (2006), suggest that object-to-location binding was specifically affected by age in comparison to trials in which those features were recalled individually.

The age-related decrement is replicated in some studies where features are required to be recalled within an object (i.e., within-object feature binding). For instance, Brown and Brockmole (2010) investigated age-related effects on binding color and shape in two experiments, and found an age-related difference for binding trials (Experiment 2; Brown and Brockmole, 2010). In line with these results, further research has also found age-related effects of feature binding of color and shapes (Brockmole and Logie, 2013; Experiment 3 of Brown et al., 2017). Additionally, binding impairments are evident in experiments probing the precision of OAs WM representations (Peich et al., 2013; Mitchell et al., 2018). In these studies, performance was measured through response dials requiring the most precise estimate of either color and orientation of previously presented colored bars at different orientations (Peich et al., 2013), or through a response color wheel that the participant had to adjust to recall the color of a previously presented probe item (Mitchell et al., 2018).

Notably, not all studies have provided positive results on aging and feature binding. Several investigations have not found a specific age-related effect on binding, either in within-object feature binding (Brockmole et al., 2008; Experiment 1 of Brown and Brockmole, 2010; Parra et al., 2009; Rhodes et al., 2016; Experiments 1 and 2 of Brown et al., 2017; Killin et al., 2018), or in object-to-location binding of complex fractals (Pertzov et al., 2015), or feature-location binding of shapes (Read et al., 2016). Similarly, in a more recent follow-up of Cowan et al. (2006) study (investigating more specifically both color and shape conjunction detection and color and location detection) Rhodes et al. (2017) found that the detection of feature binding was not disproportionately affected by aging.

One explanation proposed to account for the discrepant results is that recall of bound features declines in aging to the same extent as for individual feature recall. For instance, Brockmole et al. (2008) found that, although OAs showed significant differences between conditions of individual color recall and binding conditions, there were no significant differences between individual shape recall and binding conditions. This led the researchers to suggest that OAs difficulties arise from an impairment in maintaining shapes, not from a particular deficit in the bound representation of shape and color. Notably, this finding was also replicated by Isella et al. (2015) with a larger sample size. Brockmole and Logie (2013) and Pertzov et al. (2015) came to similar conclusions, proposing that the differential age-related effects of binding found in their study were comparable to age-related WM decline overall (Brockmole and Logie, 2013) or due to a decline in forgetting objects altogether (Pertzov et al., 2015), as opposed to a specific deficit in recalling bound representations. Further potential explanations for the discrepant findings across the studies investigating binding in healthy aging have included small sample sizes in earlier studies (see Isella et al., 2015), and the possible involvement of verbal mechanisms that OAs may recruit to compensate for declining visual WM (Forsberg et al., 2019). Forsberg et al. (2019) proposed that verbal mechanisms would be effective when a small amount of information needs to be recalled (i.e., one feature), but such compensatory mechanisms would not suffice when multiple features (i.e., bound representations) need to be recalled. Moreover, the various methodological differences across these binding studies (see Supplementary Table 1) could explain the heterogeneity of findings.

## The interaction with set size

Recent WM studies and models (Bays, 2014, 2019; see also Bays, 2015; Schneegans and Bays, 2017; Schurgin et al., 2020) have focused on aspects (such as item similarity, item familiarity, and neural noise) that converge in highlighting the role of the content of object representations to explain WM. Additionally, Bays (2014; see also Bays, 2015) and Schurgin et al. (2020) found associations between measurements of the content of object representations and set size. Altogether, the emerging view on WM in young adulthood indicates that (a) the content of the representations has a role in determining WM efficiency, and (b) the interaction between the quality and quantity of the maintained representations is a critical aspect to fully predict WM functioning.

In line with these recent accounts, we propose that assessing simultaneously the role of quantity (e.g., by means of set size) and quality (e.g., by means of feature binding) in WM performance of OAs would help unveil the nature of the age-related WM decline. However, most of the studies on age-related changes in WM have examined the impact of set size on performance (e.g., Jost et al., 2011; Sander et al., 2011; Ko et al., 2014), while only a few studies (reported above) have addressed the role of feature binding, or their interaction, in the age-related decrement (Supplementary Table 1). Among the latter ones, Cowan et al. (2006; Exp. 1a) used a varying set size of elements and found a significant interaction between age, array size and memory condition (individual item recall vs. binding conditions). OAs showed an impaired performance even for the lowest set size (four) in comparison to the individual item recall. In contrast, YAs only had a detrimental binding performance for the larger set arrays. Although these results should be interpreted with caution (see Rhodes et al., 2017), they suggest that also in YAs there is an impact of recalling fully bound representations; however, this detrimental effect is evident only when there are more items to retain. In contrast, for OAs, feature binding and set size seem to have additive effects. Indeed, the content of the representations to maintain has a detrimental effect even with a low number of items to recall - OAs may have an impaired ability to fully represent items already at low set sizes, in comparison to YAs (Cowan et al., 2006). Rhodes et al. (2017; Exp. 1) also found a significant interaction between age, set-size and memory condition (individual feature recall of color and shape vs. binding condition). For both age groups, the binding and shape conditions were more difficult (in comparison to the color condition); however, in OAs the decline in performance with item increase was less enhanced than in YAs. This was evident only when shape was the critical feature (namely, for the shape recall and binding conditions). Therefore, the authors concluded that feature binding does not consistently interact with set size in determining the performance decline of older individuals.

Although these initial findings suggest that there might not be a significant interaction between set size and binding in aging (see also Brockmole et al., 2008; Exp. 1; Mitchell et al., 2018), one should consider that to date the studies investigating feature binding abilities in aging *per se* have provided mixed results, and for this reason it is difficult to reach a definitive conclusion. Furthermore, the variations in experimental design across these studies additionally make it difficult to interpret the results. For example, Cowan et al.'s (2006) object-to-location binding included the presentation of a duplicate color in the test array, whereas individual feature trials included the presentation of a new color to the test array. This is in opposition to Brockmole and Logie (2013) in which subjects were probed to identify the color, shape and location of objects. Differences in methodologies were also present across within-feature binding studies manipulating set size (see Brockmole et al., 2008; Exp. 1 and Read et al., 2016; Exp. 2).

## Concluding remarks

As we have summarized in this mini-review, further research is warranted before making a conclusive argument for or against the presence of age-related effects on feature binding, and its interaction with set size. More in general, it remains unclear what specifically declines in aging during WM tasks, in terms of the contribution of the content and quantity of the representations to the age-related differences in performance.

Since feature binding is strongly associated with attention, one key aspect to consider is the role of attention in the age-related binding and WM decline. As attention deficits are well-documented in aging (Gazzaley et al., 2005; Craik and Bialystok, 2006; Madden, 2007), the inclusion of tasks assessing attention in feature binding in aging studies would be of utmost importance (although some studies failed to show such a link, see Brown and Brockmole, 2010). A paradigm based on the Theory of Visual Attention (TVA; Bundesen, 1990), can provide measures of speed of information processing (*C*) and visual short-term memory (vSTM capacity; *k*) using a “whole report” task (Bundesen, 1990; McAvinue et al., 2012; Wiegand et al., 2014), suggesting its applicability to being included in feature binding assessments considering the involvement of attention. In TVA-based paradigms, *C* provides an estimation of the speed of encoding items into VSTM (McAvinue et al., 2012), and accordingly, in the investigation of feature binding in aging, could provide an assessment of the speed of encoding object representations across binding and single feature trials. Additionally, *k* could provide a useful estimation of the amount of representations encoded into vSTM. Wiegand et al. (2014) found OAs with lower *C* to indicate a reduction in a neural measure associated with the prioritization of object features relevant to a task (Töllner et al., 2009). The researchers concluded declining attention abilities in aging to be associated with a slowing of object encoding. Utilizing a TVA-based paradigm could provide a useful assessment of both the processing speed of encoding object representations (*C*) and the amount encoded into vSTM (*k*), in the investigation of feature binding in aging.

A second issue for future research pertains to the neural mechanisms involved in the age-related changes in feature binding. A proposal has been discussed (Parra et al., 2009; Isella et al., 2015; Rhodes et al., 2017) theorizing object-to-location binding may be more impaired than within-feature binding in aging. Object-to-location binding has been found to involve enhanced activation of the hippocampal region (Piekema et al., 2010), an area that is known to degrade in healthy aging (Raz and Rodrigue, 2006). Indeed, the hippocampal area degrades with age more than temporal, occipital and parietal regions, which have instead been found to underlie within-feature binding in YAs (Parra et al., 2014). This is further supported by findings that OAs have shown reduced hippocampal activation in comparison to YAs when completing an object-to-location feature binding task (Mitchell et al., 2000b). However, this proposal cannot accommodate all of the existing results, as not all of the object-to-location binding studies found this type of binding to be impaired in aging (see Supplementary Table 1).

The examination of feature binding in aging is also pertinent for clinical reasons. Indeed, feature binding of WM representations has been proposed as a marker for AD (Parra et al., 2010, 2011; Cecchini et al., 2022 for a review) and for individuals at vascular risk (Bika et al., 2021). Research has found individuals with AD, and asymptomatic carriers, to indicate decreased hippocampal volume associated with object-to-location binding impairments (Liang et al., 2016). Additionally, individuals with AD, and asymptomatic carriers, have demonstrated impairments in within-feature binding (Parra et al., 2010, 2011). Evidently, the importance of examining feature binding in aging extends beyond developing a more comprehensive understanding of WM in healthy aging, and may help develop screening tools to distinguish against pathological aging.

## Author contributions

All authors contributed equally to the writing of this manuscript and approved the final version of the manuscript for submission.

## Funding

The publication fee of this article was co-funded by Fondazione Cassa Di Risparmio Di Trento e Rovereto (CARITRO).

## Conflict of interest

The authors declare that the research was conducted in the absence of any commercial or financial relationships that could be construed as a potential conflict of interest.

## Publisher's note

All claims expressed in this article are solely those of the authors and do not necessarily represent those of their affiliated organizations, or those of the publisher, the editors and the reviewers. Any product that may be evaluated in this article, or claim that may be made by its manufacturer, is not guaranteed or endorsed by the publisher.
